# Effect of thermal manipulation on the biological and mechanical characteristics of horizontal platelet rich fibrin membranes

**DOI:** 10.1186/s12903-023-03412-1

**Published:** 2023-12-01

**Authors:** Qian Wu, Shimin Yu, Yulan Wang, Xiaoxin Zhang

**Affiliations:** 1 State Key Laboratory of Oral & Maxillofacial Reconstruction and Regeneration, Key Laboratory of Oral Biomedicine Ministry of Education, Hubei Key Laboratory of Stomatology, School & Hospital of Stomatology, Wuhan University, Wuhan, 430079 China; 2 Department of Stomatology, Maternal and Child Health Hospital of Hubei Province, Tongji Medical College, Huazhong University of Science and Technology, Wuhan, 430079 China

**Keywords:** Platelet-rich fibrin, Thermal manipulation, Tensile strength, Degradation property

## Abstract

**Backgroud:**

Regardless of application scenarios, proper mechanical characteristics and degradation properties are prerequisites for horizontal platelet rich fibrin (H-PRF) to manifest its ability. Among the methods used to modify PRF, thermal manipulation is promising as it is easy to handle without adding extra additives. Yet there is no consensus on optimal temperature treatment. This study aimed to investigate the effects of heating on the biological and mechanical characteristics of H-PRF and explore the optimum heating temperature for H-PRF thermal treatment.

**Methods:**

We employed a series of temperature gradients, room temperature, 50℃, 75℃, 90℃, 105℃. The microstructure and the mechanical properties were recorded by Scanning Electron Microscope (SEM) and tensile strength tests respectively. The degradation rate of H-PRF membranes was examined by digestion assay with plasmin and trypsin. The viability of cells within H-PRF membranes and the proliferation of osteoblasts cultured with extracts from different H-PRF groups was evaluated using CCK-8 assays.

**Results:**

Compared with the nonheated group, overheated manipulation beyond 90℃ can significantly prolong the degradation properties for up to 3 to 4 weeks and enhance the mass stress of H-PRF membranes. A high-temperature treatment of 105℃ accompanied by the cell activity beneath H-PRF reduced more than half, and thus, the biological effect on human osteoblasts (hFOBs) also reduced dramatically.

**Conclusions:**

High thermal manipulation can prolong the degradation properties and enhance the mechanical properties of PRF membranes accompanied by the loss of biological effect.

## Background

Autologous venous blood contains the essential elements needed for tissue regeneration: autologous cells, growth factors and fibrinogen which can be activated to form a three-dimensional fibrin network [1]. The platelet concentrates, mainly including platelet-rich plasma (PRP), Platelet-rich fibrin (PRF) and concentrated growth factor (CGF) have gained tremendous momentum in clinical applications [2–4]. Compared to other platelet concentrate products, PRF, which is easier to prepare and has less immune rejection rate, has been widely used in a variety of clinical procedures, including ridge preservation, sinus lifting, periodontal regeneration, maxillofacial surgery, orthopedic surgery, and esthetic medicine [5–11].

For a long time, PRF has been prepared using fixed-angle centrifugation, either clinically or in the laboratory. Recently, many research demonstrated that cells accumulated in an angled fashion and aggregated in the distal wall of the centrifuge tube owing to the fixed-angle centrifuge [12, 13]. Therefore, the concept of horizontal centrifugation was proposed [14]. The horizontal platelet rich fibrin (H-PRF) produced via horizontal centrifugation showed the following characteristics compared to the PRF commonly prepared by fixed-angle centrifuges: (1) better concentration of cells including platelets and leukocytes, (2) higher structural stability, (3) more even distribution of cells, (4) prolonged release of various growth factors and cytokines, and (5) less cell damage causing by centrifugation [14].

PRF membranes have been utilized in guided tissue regeneration (GTR) and guided bone regeneration (GBR) procedures as an alternative to collagen barrier membrane or in combination with other barrier membranes to enhance the biological activity of the barrier membrane [15–18]. However, two significant drawbacks that limit PRF applications are its poor mechanical properties and rapid degradation rate. Compared to other common commercial collagen membranes usually last for 16–38 weeks, PRF membrane usually degrades within 1–2 weeks which is more difficult to meet the barrier membrane duration requirements in GBR or GTR process [19, 20]. Regardless of the application form of H-PRF membrane, it is often expected to have better degradation performance and mechanical strength during healing and regeneration to protect the regeneration area and maintain the space .

To improve the degradation property and increase the mechanical properties of PRF, some researchers have attempted to increase the degree of cross-linking of fibrin fibers within the PRF [21, 22]. After UV radiation or adding crosslinkers such as glutaraldehyde or 1-ethyl-3-(3-dimethylaminopropyl) carbodiimide hydrochloride, the degradation time of collagen was prolonged significantly [23]. However, these techniques are often cytotoxic and complicated. Therefore, enhancing the degradation property of PRF through thermal manipulation have gradually gained momentum in recent years.

The previous studies on this topic revealed that using the heat-compression treatment on the PRF membrane can be extended the degradation period to at least 3 weeks [21, 24, 25]. The Albumin gel with liquid-PRF (Alb-PRF), by first heating the upper liquid platelet-poor plasma (PPP) layer and mixing it with the liquid buffy coat zone (liquid PRF), also showed the ability to maintain volume stability in vivo after 21 days [24–26]. Compared with the addition of cross-linking agents, thermal manipulation is a relatively simple and safe clinical option to improve the mechanical properties and degradation performance of PRF membranes [23]. However, there has yet to be a detailed study about the thermal manipulation of H-PRF membranes, and most importantly, there is no consensus on the optimal heating temperature of PRF. Meanwhile, heating might cause potential damage to the live cells and denature growth factors within PRF membranes. However, the effects of healing on biological properties and mechanical properties of PRF membranes have yet to be explored.

Therefore, the aim of the present study was to find out the most suitable heating temperature for H-PRF membranes by the following studies: (1) evaluating the microstructure, mechanical properties and degradation behavior of H-PRF membranes after different heat treatments at 50℃, 75℃, 90℃, 105℃ compared with nonheated treatment, (2) investigating the biological characteristics of H-PRF membrane such as internal cell activity and ability to promote the proliferation of osteoblasts after heat treatment at 50℃, 75℃, 90℃, 105℃. The present study can provide references for temperature selection in clinical PRF membrane modification and broaden the utilization of PRF membranes.

## Materials and methods

### Preparation of H-PRF membrane

The procedures with human participants carried out in our studies were approved by the Ethics Committee of the School and Hospital of Stomatology, Wuhan University (B52/2020) and following the 1964 Declaration of Helsinki and its later amendments or comparable ethical standards. All peripheral blood samples were obtained from nine healthy volunteers who gave informed consent. All of the volunteers, aged 18–30 years without the use of anticoagulants or antibiotics for at least 3 months before blood sampling, donated five blood collection tubes (10-mL glass tubes) each time. The centrifugation protocol (10 mL, 700 g, 8 min) based on previous studies was performed using a horizontal centrifuge (Weiyin Technology Co., Ltd., Wuhan, China) [14, 27, 28]. The RCF in the protocol was defined as the relative centrifugal force on the clot. After carefully removing the red blood cell layer, the remaining H-PRF clots were compressed into standard membranes about 1 min into standardized membranes that were about 1 mm thick using metal equipment kits (Weiyin Technology Co., Ltd., Wuhan, China) as previously described [29]. All metal devices, such as surgical scissors or tweezers used in the preparation, have been sterilized with autoclave sterilization, and other inconvenient heating devices, such as heaters, have been sterilized with ultraviolet rays before using.

### Thermal manipulation of the PRF membrane

Since fibrinogen begins to denaturation around 50 °C, we set the temperature gradients corresponding to 37 °C (room temperature), 50 °C, 75 °C, 90 °C, and 105 °C to explore the optimal temperature [30]. The H-PRF membranes in the control groups were treated at room temperature. The H-PRF membranes in the test group were heated at different temperature gradients using a metal plate heater (H2O3-H, Coyote Bioscience Yixing Co., Ltd), which has a dry metal surface that can be adjusted to different temperatures. The heating groups were heated under 50℃, 75℃, 90℃ and 105℃ for 10 s on both sides, respectively.

### Scanning electron microscope

The H-PRF samples were fixed at room temperature for 4 h with 2.5% glutaraldehyde (Merck, Darmstadt, Germany) and then dehydrated with gradient ethanol (25%, 50%, 75%, 100%) for 30 min at each concentration. Afterward, the samples went through critical point dehydration and gold sputter coating. The SEM images of surfaces and cross sections of H-PRF membranes were captured using a field emission scanning electron microscope (Zeiss, Sigma) under an accelerating voltage of 20 kV and a working distance of 7 mm. The magnification ranged from 5000× to 7000× at six different field views for each sample.

### The inner cell viability of PRF membranes

To evaluate the cell viability within H-PRF membranes after heating, 0.1 g H-PRF samples from each group were fractionated into small pieces and transferred into a 6-well plate and then cultured in 1 ml Dulbecco’s modified Eagle’s medium with 10% FBS and 1% penicillin G (Thermo Fisher Scientific Inc.). It should be noted that previous experimental results indicated that cells were dragged toward the bottom of the tube after centrifugation, so the lower segment of H-PRF membranes must be retained when sampling to assess cell activity [12, 13]. At time points of day 3, 100µL Cell Counting Kit-8 solution (Dojindo, Japan) was added into each well and then incubated for 1 h at room temperature away from light. The living cell number within H-PRF membranes of each group was measured by a microplate reader (PowerWave XS2, BioTek, Winooski) at 450 nm as previously described [31].

### Mechanical strength test

The mechanical properties were measured using a tension meter (ZQ-990, ZhiQu)with a maximum load capacity of 5 N at a stretching rate of 1 mm/min under standard ambient conditions. Before stretching, the original length between the two clamps was 5 mm. The maximum force and the tensile strain at break were recorded. The stress was calculated by dividing the fracture force by the transverse section area of PRF membranes. The stress-strain curve was defined as ratio between the stress difference and the corresponding strain difference. Each group in this study was repeated with three replicates.

### Cell culture

Human osteoblasts (hFOBs) were purchased from VectorScient (LSMCE022) and cultured in Dulbecco’s modified Eagle’s medium with 10% FCS and 1% antibiotics (Gibco, Thermo Fisher Scientific). The cell culture medium was changed every 2 days throughout the cell culture experiments. All subsequent cell counting procedures were performed using Countess Invitrogen 3 (Thermo Fisher Scientific).

### Cell proliferation assay

To prepare the H-PRF extracts, each treated H-PRF membrane was incubated with 5 ml DMEM (Dulbecco’s modified Eagle’s medium) supplemented with 10% FCS (fetal calf serum) and 1% penicillin G (Thermo Fisher Scientific Inc.) in a 37℃ incubator for 3 days as previously described [32]. Then, the medium was collected, filtered with 0.22 μm membrane filter (Millex-GP, PR05538, Merck Millipore Ltd.) and stored at 4℃ for future cell experiments.To avoid the insignificant differences between the groups due to excessive cell proliferation in the later stage, human osteoblasts (hFOBs) suspension was counted by Invitrogen Countess 3 (Thermo Fisher Scientific) and then diluted to 1,0000 cells per milliliter and then seeded 100 µL/well in 96-well plates as previous study recommended [32]. For the test, cells were seeded at a density of 1000 cells for proliferation experiments in 96-well plates at 37℃ with DMEM containing 10% FCS and 1% penicillin G supplemented with 20% PRF extracts from different groups (Thermo Fisher Scientific Inc). For the control group, cells were incubated with normal DMEM containing 10% FCS and 1% penicillin G. Cell culture medium was refreshed daily throughout the cell culture experiments in all groups. At 1, 3 and 5 days, 10 µL of CCK8 solution was added to each well and incubated for 30 min at 37℃ in the dark. The medium was then pipetted and quantified using a luminescence plate reader (TECAN Infinite 200 Pro) at 450 nm.

### In vitro degradation test

After rinsing with phosphate buffered saline (PBS; 150 mM NaCl, 20 mM sodium phosphate, pH 7.2) three times for 3 min to eliminate serum as possible, 0.5 g of H-PRF samples were prepared and inserted in 12-well plates and incubated in a CO_2_ incubator with 0.25% trypsin (Biofroxx, 1004GR025, PBS; 700 µM EDTA) solution in vitro. 50 mL of the digestion solution was collected every 1 h and was stored at -20℃. The BCA protein assay kit (Takara Bio, Kusatsu, Japan) was used to evaluate the concentration of digested protein from PRF membranes. The protein levels at the time of complete digestion of the initial fibrin were estimated at 100%. An equal amount of trypsin solution was added after each collection of digestion solution to maintain consistency. In order to simulate the condition in vivo, we also tested the degradation rate treated with DMEM supplemented with 20% human plasmin (Hematologic Technologies, Essex Junction, VM). The appearance and weight were photographed and recorded every day.

### Statistical analyses

GraphPad Prism software 8.0.2 was utilized to analyze the data. To test the significance of the observed differences between groups, the data were analyzed using one-way analysis of variance (ANOVA) for the inner cell viability of PRF membrane evaluation, mechanical strength test, and cell proliferation assay. Two-way analysis of variance (ANOVA) was utilized for the PRF quality measurements and the in vitro degradation test and significant differences between groups were determined by post-hoc Tukey’s multiple comparison tests. *P < 0.05, **P < 0.01, ***P < 0.001 was considered statistically significant. Each experiment was performed with at least three replicates in all experiments.

## Results

### Microstructure of H-PRF membranes

**Fig. 1:**
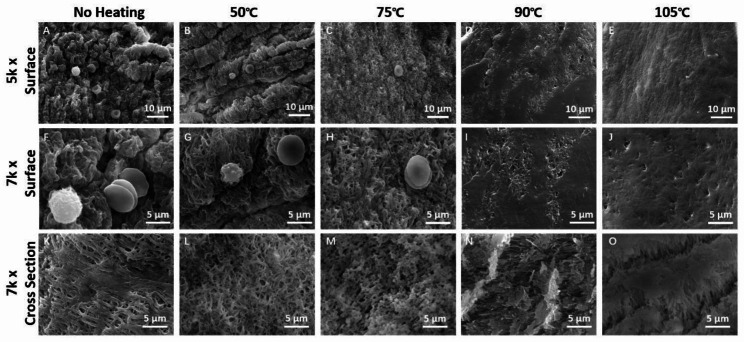
The scanning electron microscopy (SEM) images of H-PRF membranes after various heating treatments with different temperatures. **A,F,K**) H-PRF membranes without heating. **B,G,L**) H-PRF membranes heated under 50℃. **C,H,M**) H-PRF membranes heated under 75℃. **D,I,N**) H-PRF membranes heated under 90℃. **E,J,O**) H-PRF membranes heated under 105℃. The surface of each H-PRF membrane was scanned using 5k× and 7k× magnifications. The cross-sections of the samples were monitored using 7k× magnification

After different thermal manipulations, the microstructure observation of the surfaces and cross-section of each H-PRF membrane was performed by scanning electron microscopy (SEM). With the increases of temperature, the fibrin network in H-PRF became denser and the number of visible cells within the H-PRF membrane was less than the control group (Fig. 1). When treated at temperatures above 90℃, almost no living cells and normal fibrous network were observed, and the membrane seemed flat and compact. The changes of the fibrin network were more obvious in cross-section. In the unheated group, the fibrin network was uniformly dispersed. With the increase of temperature, the fibrin network began to adhere, and the voids decreased. At the 105 °C group, almost no regular fiber voids could be observed instead of some cracks caused by tearing.

### Mechanical strength of PRF membranes after heat treatment

**Fig. 2:**
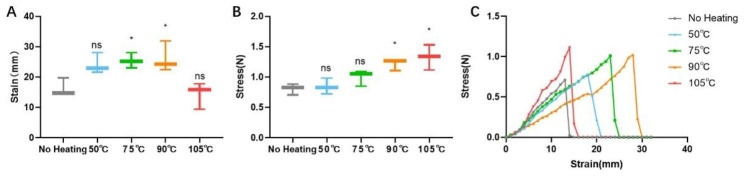
The various mechanical properties of H-PRF membranes after different temperature heat treatments. **(A)** The tensile strain at break of H-PRF membranes following various heat treatments. **(B)** The maximum stress force of H-PRF membranes under different heat treatments. **(C)** Representative stress-strain curves of H-PRF membranes after heat treatments at various temperature. Each group was analyzed with three replicates. Statistical differences were compared between the control non-heated H-PRF group and the other heating groups. ns: not statistically significant versus control group; *P < 0.05, **P < 0.01, and ***P < 0.001

The mechanical properties of H-PRF membranes were characterized by the maximum stress, the tensile strain at break and the stress-strain curves. The maximum stress is defined as dividing the force by the initial cross-sectional area. The tensile strain at break and the strain-stress curve can reflect the elasticity of the H-PRF membranes. The stress-strain curve was defined as ratio between the stress difference and the corresponding strain difference. The strain at break of the H-PRF membranes were significantly improved as the temperature rose but suddenly dropped at 105℃, which represented the overheating led to the decline of elasticity (Fig. 2A). The maximum stress in all heated groups was higher than that in non-heated group and increased with the rises of temperature (Fig. 2B). The stress-strain curve showed that H-PRF membranes in the control group could be stretched 1–2 times their original length, while the heated groups under 90℃ could be stretched up to 2–3 times their initial length until rupture (Fig. 2C). With the dramatic drop of the tensile strain at break in the treated group under 105℃, its stress-strain became narrower and steeper compared to other groups, which indicated that the H-PRF under overheated treatment might be hard and brittle.

### Internal cell viability of H-PRF membranes after heat treatment

**Fig. 3:**
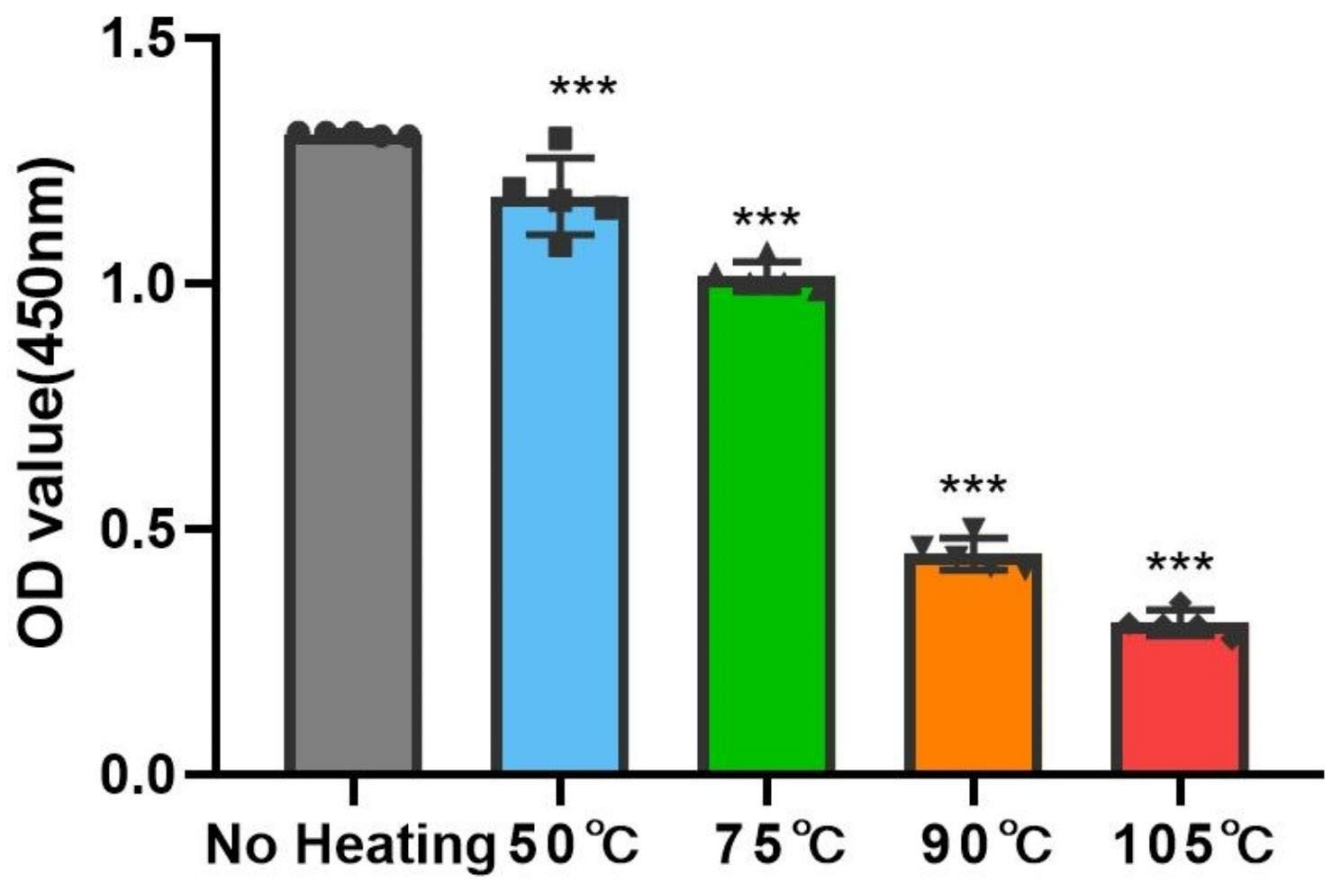
The results of using CCK8 test to evaluate the survival condition of cells within the H-PRF membranes under different heat treatment. Each group was analyzed with five replicates. Statistical differences were compared between the control non-heated H-PRF group and the other heating groups. ***P < 0.001

We then evaluated the viability of cells within the H-PRF membranes under various heat treatment modalities through CCK-8 assays. All of the heated groups showed a decrease in internal cell activity compared to the control non-heated group. With the increase in temperature, the active cells within H-PRF membranes decreased gradually (Fig. 3). It is noteworthy to point out that the OD value under 450 nm in the 90℃ and 105℃ heated groups showed a dramatic reduction of more than two-thirds compared with the non-heated group, confirming the activity of the cells within H-PRF was greatly damaged through overheating.

### Degradation of H-PRF membranes

**Fig. 4:**
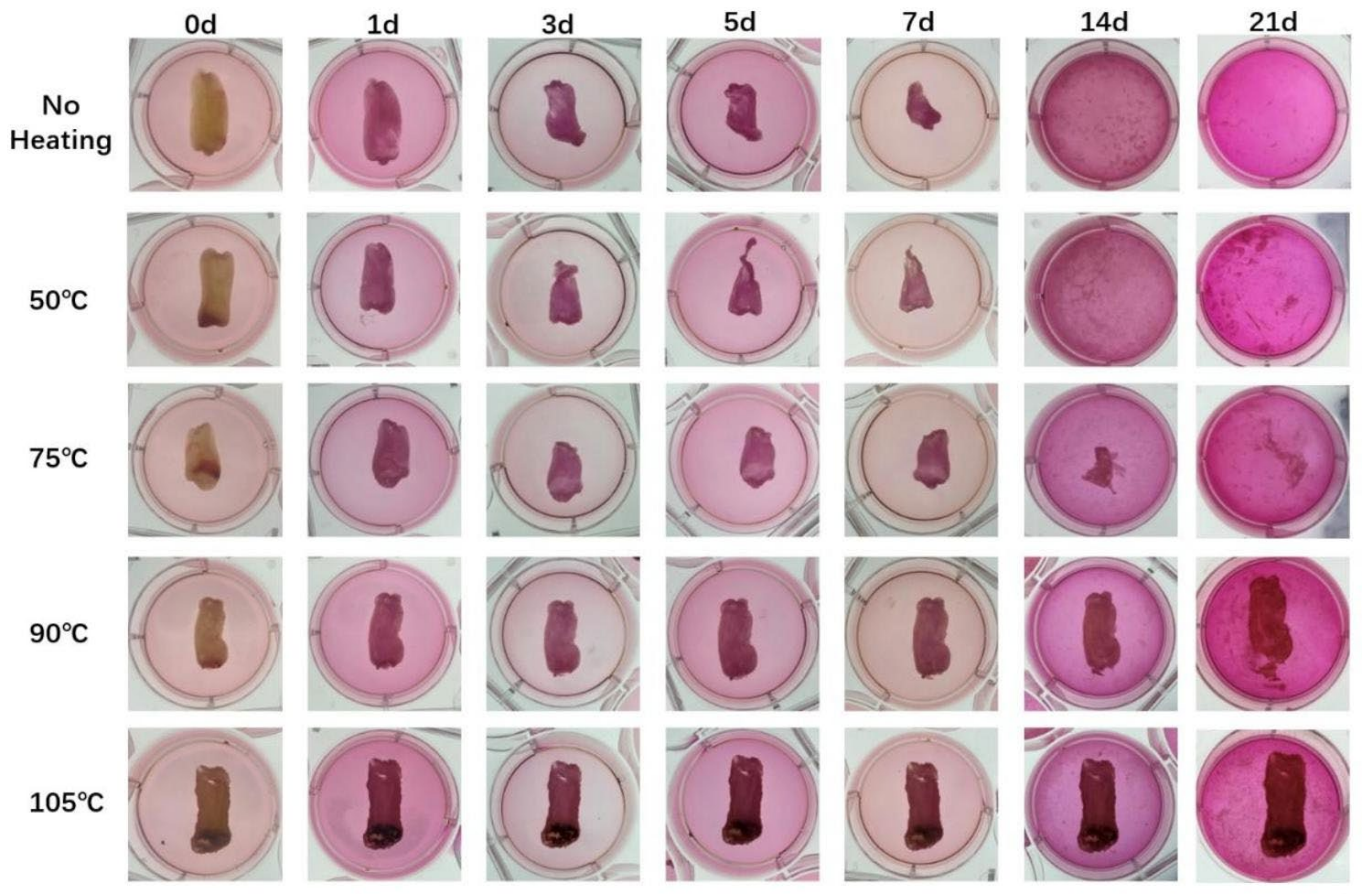
Representative time-course degradation of H-PRF membranes following different heat treatment. The H-PRF membranes were cultured in Dulbecco’s modified Eagle’s medium with 10% FCS and 1% antibiotics (Gibco, Thermo Fisher Scientific). Each group was analyzed with three replicates

**Fig. 5:**
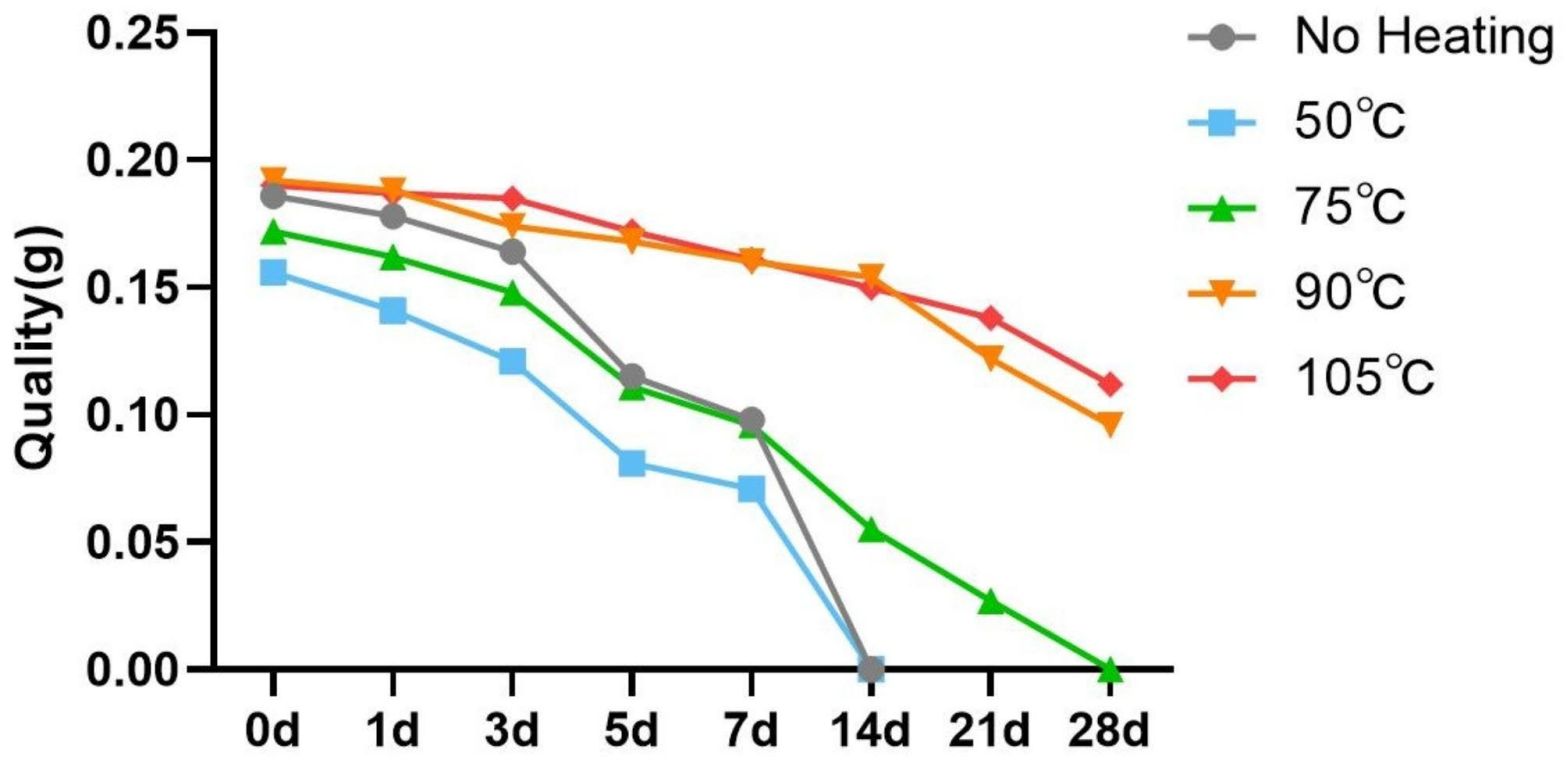
The quality changes of different groups of H-PRF membranes. All the H-PRF membranes were cultured in Dulbecco’s modified Eagle’s medium with 10% FCS and 1% antibiotics (Gibco, Thermo Fisher Scientific). Each group was analyzed with three replicates

**Fig. 6:**
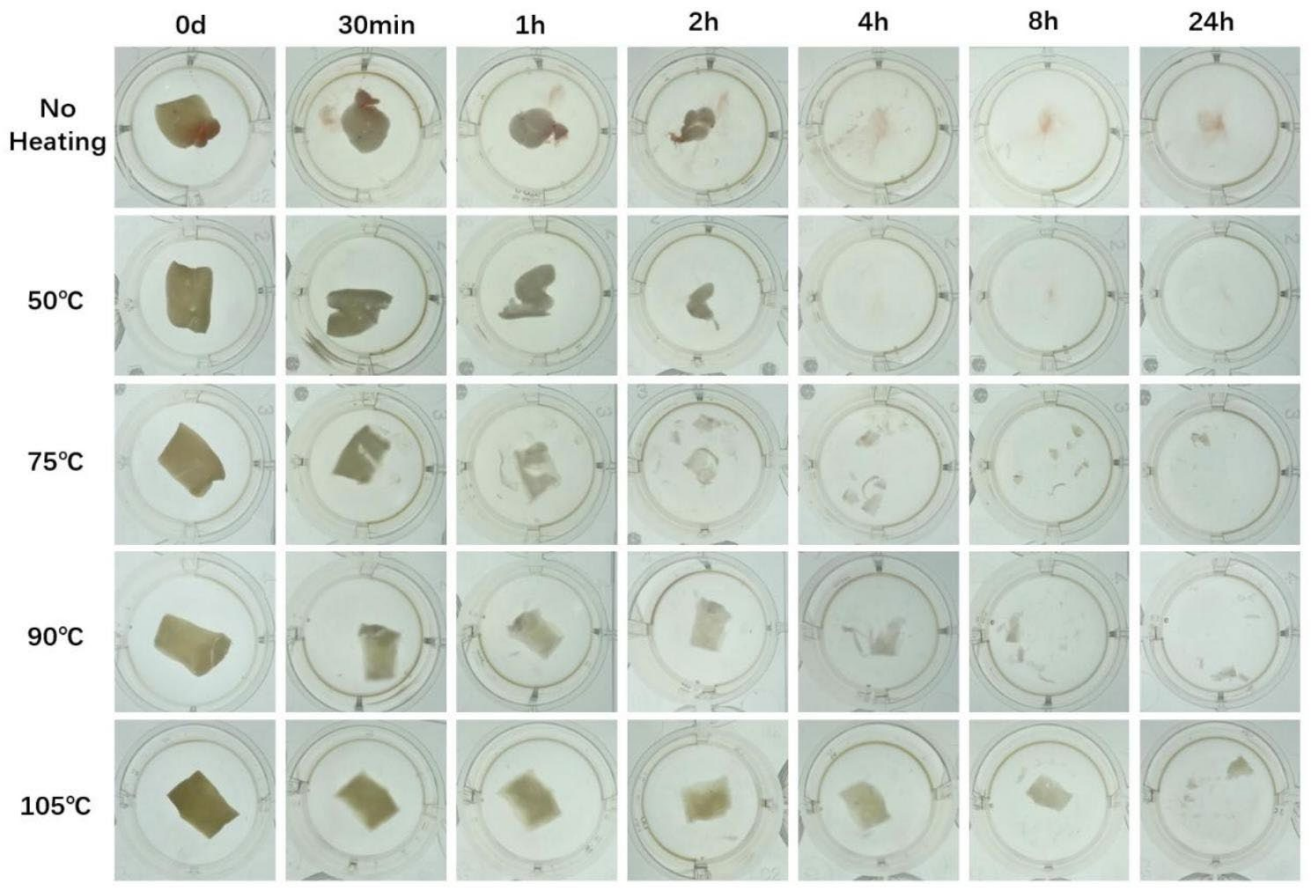
Representative time-course degradation of H-PRF membranes following different heat treatment. The H-PRF membranes were cultured in PBS with 0.25% trypsin. Each group was analyzed with three replicates

**Fig. 7:**
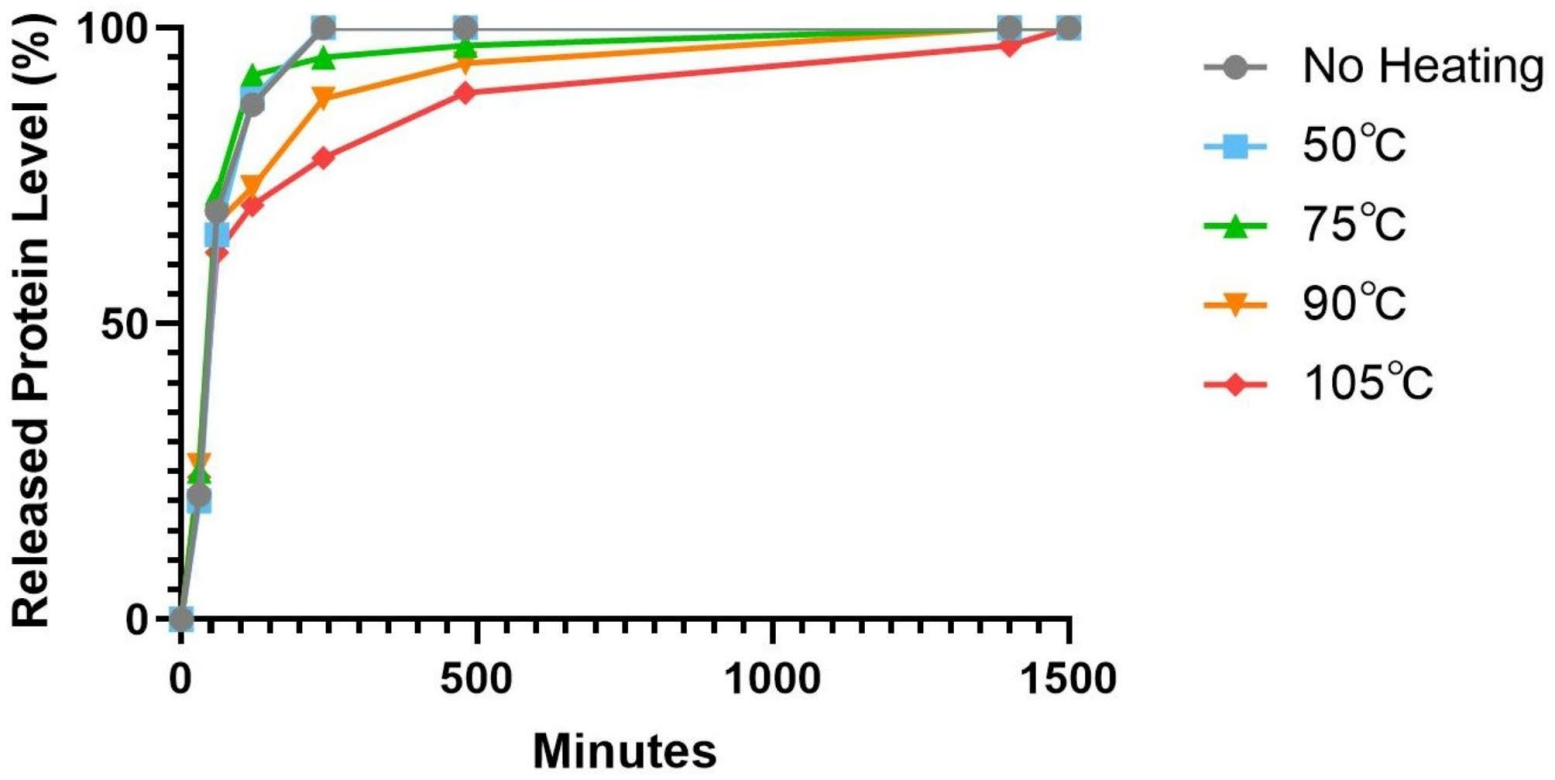
Enzymatic degradability of H-PRF membranes following different heat treatment. The H-PRF membranes were cultured in PBS with 0.25% trypsin. Protein levels were determined by a BCA protein assay kit (Takara Bio, Kusatsu, Japan)

The H-PRF membrane degradation test was carried out in a serum medium to simulate the degradation of H-PRF in vivo. The degradation rate of H-PRF membranes in the unheated and 50℃ heating groups was significantly faster than that in other heating groups, and all the membranes were completely degraded within 2 weeks.In comparison, the H-PRF membranes in the 75℃ heat treatment group degraded in approximately 3 weeks, while the PRF membranes using the 90℃ and 105℃ heat treatment lasted for more than 3 to 4 weeks of incubation (Figs. 4 and 5). Since H-PRF is mainly composed of protein components, 0.25% trypsin was also used for degradation assay, and the medium was collected and measured protein content at each time point by the BCA testing kit. Most of the H-PRF in nonheated and 50℃ heated groups was degraded completely within 4 h co-culture with 0.25% trypsin. Most of the H-PRF membranes in the 75℃ and 90℃ heated groups were degraded mostly within 8 h, while the H-PRF membranes in the 105℃ heated group still retained a relatively complete form, and degraded completely after 24 h incubation (Figs. 6 and 7).

### Influence of H-PRF membranes with different heat treatments on human osteoblast proliferation

**Fig. 8:**
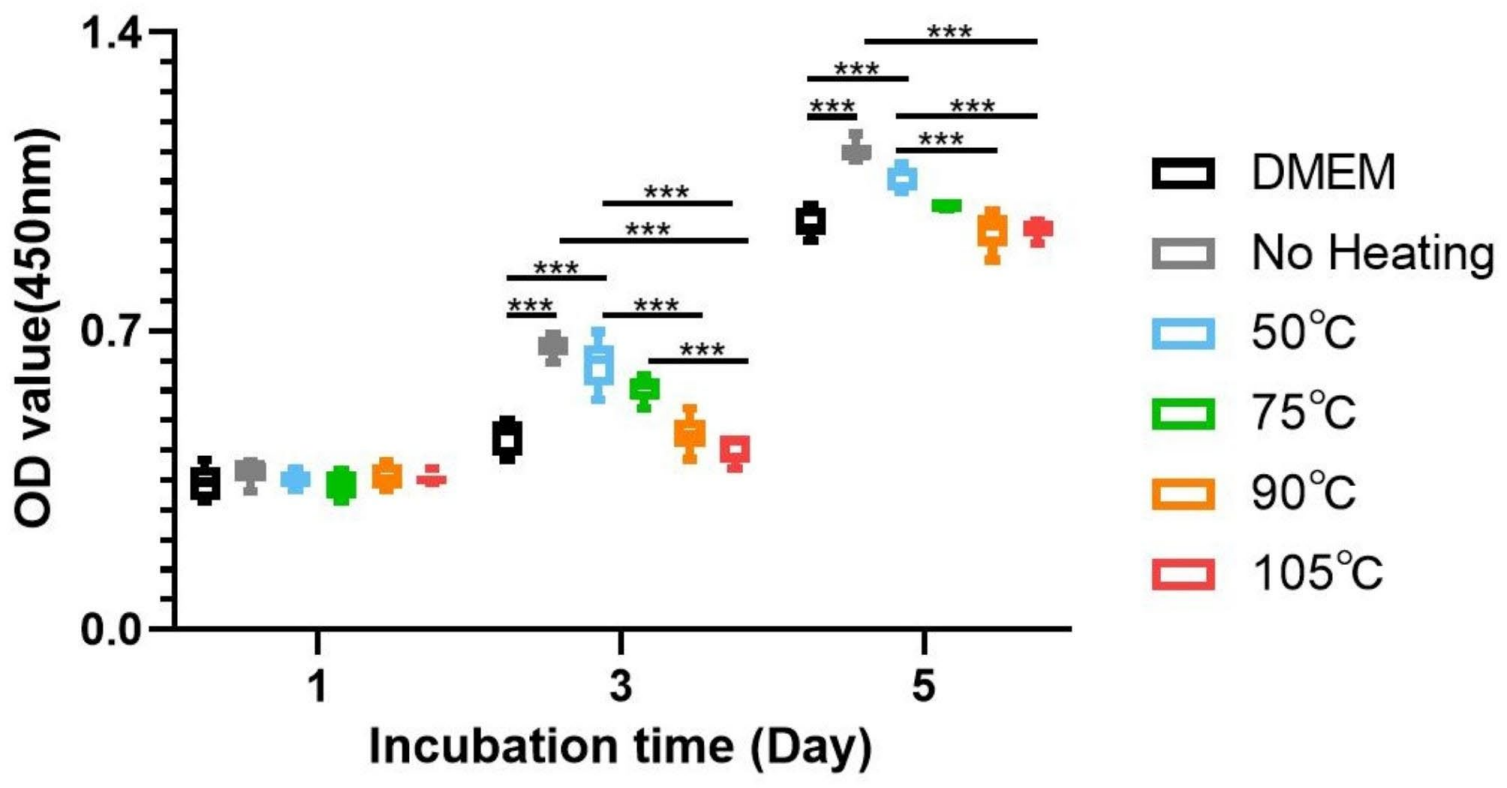
The proliferation rates of human osteoblasts cultured with DMEM containing 10% FCS and 1% penicillin G supplemented with 20% PRF extracts from different groups. CCK8 assays were performed at 1, 3 and 5 days post-seeding. ***P < 0.001

For wide application in guided bone regeneration procedures, the impact on the proliferation capacity of osteoblasts is one of the essential properties of H-PRF membranes. Human osteoblasts were cultured with DMEM containing 10% FCS and 1% penicillin G supplemented with 20% PRF extracts from different groups. Then the cell proliferation capacity on days 1, 3, and 5 was investigated using CCK-8 assays (Fig. 8). It was demonstrated that no significant difference was observed between all groups on day 1, which may reflect that the initial cells’ distribution was relatively even in different groups because osteoblasts were just mainly attached to the bottom of the 96-well plates 1 day after colonization. However, on days 3 and 5, it was found that cells cultured with the nonheated H-PRF showed significantly higher cell numbers compared to the blank DMEM group, which demonstrated the active cells and growth factors within H-PRF are benefit for the proliferation of osteoblasts. With the increase of temperature, the effect of PRF on the proliferation of osteoblasts decreased gradually. Notably, the heat treatment group under 90℃ still demonstrated a proliferative increase in cell numbers compared to the blank group, confirming remaining bioactivity ever after thermal manipulation. However, in the 90℃ and 105℃ heated group, the bioactivity of H-PRF decreased significantly, and there was no significant difference inthe ability to promote osteoblast proliferation between their exudate and pure DMEM.

## Discussion

Recent studies about PRF modification by heating mainly focus on the change of degradation performance, and there is no consensus on the optimal temperature, and a detailed evaluation of the biological and mechanical characteristics of H-PRF at different temperatures is also lacking. In our study, we set up different temperature groups at 50℃, 75℃, 90℃ and105℃. Then the biological and mechanical properties of H-PRF were investigated comprehensively.

The microstructure of H-PRF was observed by SEM. Fibrinogen is heat sensitive andprecipitates above 56℃ [30]. Thus, by scanning electron microscopy, uniform fibrin networks can be found in the unheated group and the fibrin structure seemed to be maintained in the heating mode below 50℃.At the same time, obvious differences were observed between 90℃ and 105℃ heated groups, the pores between the fibrin bundles appeared to shrink significantly. The number of intact cells observed also significantly decreased due to the high processing temperatures. This result may be related to subsequent changes in mechanical properties, and the reduction of the porosity of the fibrin network may have a more substantial isolation effect on bacteria or cells. The heated group may be able to better isolate the external soft tissue into the bone defect area in the GBR process. Although the mechanical properties test results showed that the maximum stress of H-PRF membranes increased with the temperature, the tensile strain at break suddenly decreased at 105℃. It may indicate that after excessive heating, H-PRF membranes seem to be more solid, but its toughness and elasticity decreased. The results may be related to the denaturation of fibrin at high temperature.For clinical applications, the heated group of PRF membranes seems to obtain greater strength and better avoid perforation or collapse caused by tissue tension or compression.

In the present study, to further simulate the situation in vivo, we evaluated H-PRF membrane interpretation in serum-containing media. Since H-PRF contains a three-dimensional protein network, we treated it with trypsin and observed its degradation using the quantitative BCA test. The results of two different degradation experiments showed that the heated treatment can effectively prolong the degradation time of H-PRF membranes both in serum and in trypsin conditions. During the thermal manipulation process, it is demonstrated that the secondary structure of the albumin protein was modified, and new hydrogen or ligations were formed sequentially, which might contribute to prolonging the degradation rate and improving the stability of H-PRF [23]. The internal cell viability test did prove that high temperature caused cells within the H-PRF membrane death and reduced its activation behavior to osteoblasts. These results were consistent with the above scanning electron microscopy (SEM) images, which showed that heating resulted in increased mechanical properties and prolonged degradation, but damaged active cells within the plasma matrix.

This article may provide some reference for our clinical operation. For example, it is trendy to cover H-PRF membranes above the titanium mesh to use the active leukocytes inside it to reduce the risk of exposure to titanium mesh and resist bacterial infection. The high-temperature modes are unsuitable while the clinicians look for more cell activity. However, clinicians can replace the collagen membrane with the H-PRF membrane after thermal treatments in some defect areas. Then clinicians can cover it with the H-PRF membrane after unheated or low-temperature treatment to complement their advantages and disadvantages. The subsequent loading of drugs or growth factors may compensate for the effect of high temperature treatment on H-PRF activity [33].

### Limitations of the study

It is ideal for clinicians to use the patient’s own blood to prepare the PRF membrane with excellent strength and degradation performance through simple heating treatment. However, the loss of active cell components caused by heating is also regrettable. Therefore, more in vivo studies and clinical effects trials are required to validate this new regeneration technique further.

## Conclusion

In conclusion, the present study evaluated the mechanical and physical properties, internal cell activity and bioactivity of H-PRF membrane to osteoblasts under different heating temperatures. High thermal manipulation beyond 90℃ can significantly prolong the degradation properties and enhance the mass stress of PRF membranes, accompaniedby the loss of active cells and biological effect. Clinicians need to choose appropriate treatment methods in clinical therapies according to different clinical application scenarios.

## Data Availability

The datasets used and analysed during the current study available from the corresponding author on reasonable request.

## List of abbreviations

H-PRF: Horizontal Platelet Rich Fibrin
SEM: Scanning Electron Microscope
CCK−8 test: Cell Counting Kit−8 Test
hFOBs: Human Osteoblasts
PRP: Platelet Rich Plasma
PRF: Platelet Rich Fibrin
CGF: Concentrated Growth Factor
GTR: Guided Tissue Regeneration
GBR: Guided Bone Regeneration
UV: Ultraviolet
Alb-PRF: Albumin Gel with Liquid-PRF
FBS: Fetal Bovine Serum
DMEM: Eagle’s Minimal Essential Medium
PBS: Phosphate-Buffered Saline
EDTA: Ethylenediaminetetraacetic Acid
BCA: Bicinchoninic Acid Assay
ANOVA: Analysis of Variance
